# Optimizing Humoral Immunity for Durable and Broad Protection in Flavivirus Vaccines

**DOI:** 10.3390/vaccines13121182

**Published:** 2025-11-21

**Authors:** Jae-Yeon Park, Hye-Mi Lee

**Affiliations:** College of Veterinary Medicine, Chungnam National University, Daejeon 34134, Republic of Korea

**Keywords:** flavivirus vaccination, humoral immunity, antibody quality, cross-protection, adjuvant design, vaccine durability

## Abstract

Flavivirus infections, including dengue, Zika, West Nile, and Japanese encephalitis, remain a major global health concern. Although several vaccines are licensed, the durability and qualitative features of vaccine-induced antibodies differ substantially across platforms, leading to incomplete cross-protection and the risk of antibody-dependent enhancement. Long-term durability is exemplified by YF-17D, which induces protective antibodies that have been detectable for decades, whereas the JE SA14-14-2 vaccine has achieved program-level reductions in disease in endemic regions. In contrast, CYD-TDV shows serostatus-dependent outcomes, and the investigational TAK-003 vaccine has demonstrated antibody persistence for at least four years. Recent studies have clarified how preserving quaternary envelope epitopes, minimizing prM-associated non-neutralizing specificity, and sustaining germinal center activity determine antibody affinity, breadth, and persistence. Advances in adjuvant formulations and delivery platforms have shown that engaging defined innate pathways and prolonging antigen availability enhance affinity maturation and long-lived plasma cell formation. Booster scheduling and baseline serostatus further shape the antibody quality, highlighting the importance of immune imprinting and cross-reactivity in vaccine design. Together, these findings outline the design principles for next-generation flavivirus vaccines, including stabilization of neutralization-sensitive epitopes, use of adjuvants that sustain germinal center responses, optimization of antigen persistence, and tailoring of dosing strategies to immune history to elicit durable and broadly protective humoral immunity.

## 1. Introduction

Flaviviruses are small enveloped positive-sense single-stranded RNA viruses belonging to the family Flaviviridae, which includes dengue, Zika, West Nile, yellow fever (YF), and Japanese encephalitis (JE) [1,2]. They continue to cause large outbreaks across tropical and temperate regions, such as human mobility, climate trends, and vector adaptation, thereby expanding transmission zones worldwide [3]. The virion encodes three structural and seven nonstructural proteins through a single open reading frame that coordinates entry, genome replication, assembly, and immune evasion, creating multiple points at which host immunity and vaccines can act [4,5,6]. The envelope glycoprotein mediates receptor binding and low pH-triggered membrane fusion and is the dominant target of neutralizing antibodies elicited by infection or vaccination [7]. The global disease burden remains high, with dengue alone causing hundreds of millions of infections annually, and with an expanding geographic footprint documented in recent mapping studies [8,9].

Vaccination demonstrates the potential for humoral protection in this genus. The YF vaccine elicits durable immunity, and JE vaccination programs have significantly reduced its incidence in regions with sustained coverage [10,11]. Population-level assessments further confirmed these reductions following the national program implementation [12,13]. Despite these successes, vaccines for dengue, Zika, and West Nile remain limited or only partially protective because antigenic diversity and immune cross-reactivity complicate the definition of reliable correlates of protection across exposure and age group [14,15,16].

Humoral immunity is the primary cause of protection against flavivirus infection and vaccination. Neutralizing antibodies targeting envelope (E) proteins block attachment and fusion to prevent systemic infections [17,18]. Antibody concentration alone does not predict vaccine performance, since qualitative features including affinity, avidity, epitope specificity, isotype distribution, and persistence of long-lived plasma cells and memory B-cells determine the durability and effectiveness of protection in real-world settings [19,20,21,22].

Cross-reactive antibodies are a hallmark of flavivirus immunity and may protect high titers. At intermediate titers, they can facilitate infection through antibody-dependent enhancement, as demonstrated in a long-term pediatric surveillance study that identified a narrow range of pre-existing titers associated with a maximal risk of severe dengue [23,24]. Heterologous interactions between flaviviruses are also relevant. Prior dengue infection generates antibodies that bind Zika virus (ZIKV) with avidity and modulate subsequent immune and clinical outcomes during sequential epidemics in endemic regions [25].

Although key aspects of T cell immunity are referenced where directly relevant to germinal center activity and affinity maturation, a detailed analysis of cellular correlates is outside the scope of this review. The primary focus is on E protein-directed humoral immunity, including the structural, adjuvant, and platform-level determinants that shape antibody specificity, avidity, breadth, and longevity. Structural virology has identified conserved epitopes on the envelope dimer interface that are recognized by broadly neutralizing human antibodies. These epitopes provide molecular templates for immunogen design aimed at increasing breadth while reducing non-neutralizing specificity [26,27]. Adjuvant research has shown that pattern recognition receptor agonists, saponin-based formulations, and optimized emulsions promote higher quality and more durable antibody responses by enhancing germinal center activity and T follicular helper (Tfh) cell support, surpassing the effects of alum [28,29]. Platform advances have contributed to this trend. mRNA vaccines and nanoparticle-based delivery systems prolong antigen availability and improve antigen display, thereby strengthening the magnitude, quality, and persistence of antibody responses in preclinical and clinical studies [30,31]. Taken together, the current evidence indicates that improving antibody quality and durability is essential for achieving broad and long-lasting protection against flaviviruses. These insights support integrated vaccine strategies that combine rational adjuvant selection, epitope-focused antigen engineering, and advanced delivery platforms to elicit high-affinity, cross-protective, and persistent humoral immunity, while maintaining safety across varied exposure histories and populations. Collectively, these observations converge on a set of design principles that guide durable and broadly protective flavivirus vaccine immunity.

1. Preserving quaternary E-dimer epitopes to promote potent and broad neutralization while minimizing off-target responses;
2. Reducing prM-associated or fusion loop–biased non-neutralizing specificities that contribute to ADE risk;
3. Sustaining germinal center activity and Tfh support to drive affinity maturation and long-lived plasma cell development;
4. Optimizing antigen persistence and delivery platform properties to reinforce durable and high-avidity antibody responses;
5. Incorporating baseline serostatus and immune imprinting patterns into vaccine design can ensure safety and consistent immunogenicity across populations.

These principles serve as the organizing thread for Section 3, Section 4, Section 5, Section 6 and Section 7 and are revisited in the conclusion to maintain a unified conceptual backbone throughout the manuscript.

## 2. Overview of Humoral Immunity in Flavivirus Vaccination

Humoral immunity is the principal correlate of protection against flavivirus infections and is a major determinant of vaccine performance. Following vaccination, B-cell activation, germinal center maturation, and progressive antibody affinity improvement collectively define the quality and durability of neutralizing responses. Comparative data from licensed vaccines, including those against YF, JE, and dengue, revealed distinct profiles of antibody magnitude, persistence, and functional characteristics (Table 1). Understanding these processes provides a mechanistic foundation for interpreting vaccine-induced immunity across different platforms.

### 2.1. B-Cell Activation and Germinal Center Response

Effective flavivirus vaccines initiate a coordinated sequence of immune events, beginning with B-cell activation. Naïve B cells that recognize conformational epitopes on the viral E protein are activated through B-cell receptor engagement and Tfh cell assistance [22,32]. Within the germinal centers of draining lymph nodes, activated B-cells undergo proliferation, somatic hypermutation, and class-switch recombination processes that refine antibody specificity and effector potential [22,33].

Adjuvant and live-attenuated vaccine platforms strongly influence germinal center kinetics. The live-attenuated YF-17D vaccine induces extended germinal center activity and generates an antibody repertoire that has persisted for decades [34,35]. Similarly, the live-attenuated SA-14-14-2 JE vaccine elicits robust B-cell activation and durable memory responses, whereas inactivated formulations require periodic boosting to maintain sero-protection [13,36]. Adjuvants such as alum primarily increase antibody magnitude, whereas saponin-based or pattern recognition receptor agonist formulations promote more sustained germinal center activity and support higher antibody quality [28,29]. Collectively, these observations highlight that germinal center dynamics shape not only the antibody titer but also the breadth and persistence of protection.

### 2.2. Antibody Affinity Maturation and Long-Lived Plasma Cell Formation

Affinity maturation occurs through iterative selection of B-cell clones with improved immunoglobulin receptors, driven by activation-induced cytidine deaminase (AID)-mediated somatic hypermutation [37,38]. This process produces high-affinity antibodies that efficiently recognize quaternary epitopes on the envelope protein, improving neutralization potency and cross-serotype breadth [27,39].

Following the germinal center phase, a subset of B-cells differentiate into long-lived plasma cells that migrate to the bone marrow and continuously secrete antibodies for extended periods [40,41]. The maintenance of these plasma cells depends on stromal cell-derived survival factors, such as interleukin (IL)-6, B-cell-activating factor (BAFF), and APRIL, which sustain antibody production even after antigen clearance [42].

Among licensed flavivirus vaccines, YF-17D generates one of the most durable antibody responses, with neutralizing activity persisting for more than 30 years after a single dose [34,43]. In contrast, immunity following inactivated JE vaccines wanes within several years and requires booster doses to maintain protection [13,44]. These differences illustrate how antigen persistence and vaccine platform characteristics influence the development and longevity of plasma cells and memory B-cell compartments [30,32].

### 2.3. Neutralizing and Non-Neutralizing Antibody Functions

Neutralizing antibodies protect the virus primarily by blocking viral attachment, membrane fusion, and post-entry processes [45,46]. Structural analyses of broadly neutralizing antibodies (bnAbs) often recognize quaternary epitopes bridging adjacent E protein dimers, such as the envelope dimer epitope (EDE), which mediates cross-neutralization among dengue serotypes and partial activity against ZIKV [26,27].

Non-neutralizing antibodies contribute to antiviral defense by engaging Fc-gamma (Fcγ) receptors on immune cells and promoting antibody-dependent cellular cytotoxicity and phagocytosis [47,48]. At suboptimal antibody concentrations, Fc-mediated uptake can facilitate viral replication in monocytes and macrophages, resulting in antibody-dependent enhancement (ADE) [23,49].

Therefore, balancing neutralizing potency with Fc-mediated effector function is essential for flavivirus vaccine safety. Comparative serological analyses of licensed vaccines showed that YF and JE vaccines induce strong neutralizing antibodies with a likelihood of ADE, whereas dengue vaccines generate broader but more heterogeneous responses that require careful evaluation of the cross-reactive antibody quality [13,43,50]. These distinctions are summarized in Table 1, which compares the magnitude, breadth, and durability of antibody responses among licensed flavivirus vaccines and highlights how the vaccine platform, antigen conformation, and adjuvant formulation shape protective humoral immunity.

**Table 1:** Comparative antibody responses to licensed flavivirus vaccines.

Vaccine	Platform	Neutralizing Magnitude	Durability Tier	Breadth/Cross-Reactivity	ADE/Safe	Refs.
YF-17D	Live-attenuated	High titers within 2–4 weeks; polyfunctional IgG	>20 years in clinical follow up	Cross-reactive within YF genotypes; minimal heterologous activity	No ADE signal reported	[34,43]
JE-SA-14-14-2	Live-attenuated	Robust titers after single dose; strong memory recall	>20 years in clinical follow up	Limited cross-neutralization to other JEV genotypes	Well-tolerated; no ADE	[13,44]
JE (IXIARO/inactivated)	Purified inactivated	Moderate titers; boosted by 2-dose regimen	wanes over 2–3 years	Genotype-specific; minimal heterologous response	no ADE	[44]
Dengue (CYD-TDV)	Chimeric yellow-fever vector	Strong neutralization; dependent on serostatus	wanes over 2–3 years	Broad but heterogeneous; partial neutralization of heterologous serotypes	Increased ADE risk in sero-negatives	[23,24,50]
Dengue (TAK-003)	Live-attenuated tetravalent	Balanced neutralization across serotypes	≥4 years in clinical trials so far	Cross-neutralization with modest enhancement potential	No ADE signal to date	[51]
Zika (mRNA/DNA candidates)	Nucleic acid (investigational)	High titers in animals and early human trials	anticipated from platform mechanism	Strong cross-reactivity with DENV; epitope overlap	ADE potential in vitro; not confirmed in vivo	[30,52]

Durability descriptors follow the standardized terminology used throughout the manuscript. Evidence tiers are harmonized using the categories Phase 3 clinical data, preclinical murine or NHP data, observational serology, and mechanistic inference. These terms match the terminology used throughout this manuscript. IgG, immunoglobulin G; ADE, antibody-dependent enhancement.

## 3. Influence of Adjuvants on Antibody Quality and Durability

Adjuvants profoundly shape the magnitude, quality, and persistence of vaccine-induced antibody responses by modulating innate sensing, antigen presentation, and germinal center formation dynamics. Their mechanisms determine whether the resulting antibodies exhibit sustained affinity maturation, long-term plasma cell support, and cross-serotype breadth [53,54]. Differences in adjuvant formulations accounted for most of the variation in antibody durability and breadth observed across vaccine platforms. Classical adjuvants, such as alum or oil-in-water emulsions, have enhanced early protective titers for decades, but these formulations show limited ability to sustain germinal center activity compared to systems that engage pattern-recognition receptors or the STING pathway. These adjuvant-driven effects align with the third design principle, which emphasizes the need to sustain germinal center activity and Tfh support to generate high-avidity antibodies and durable, long-lived plasma cell responses. To maintain a consistent and parallel structure across adjuvant classes, the subsections that follow are organized along four dimensions: enhancement of germinal center and Tfh activity, influence on IgG subclass or antibody avidity, expected durability of the antibody response, and representative flavivirus vaccine applications.

### 3.1. Classical Adjuvants and Their Immunomodulatory Effects

Alum, the oldest and most widely used adjuvant, improves antigen retention at the injection site and recruits monocytes and dendritic cells via local inflammasome activation [55]. This activation triggers IL-1β and IL-18 release, leading to efficient priming of naïve B-cells but relatively modest Tfh cell differentiation, which constrains affinity maturation and accelerates antibody waning [53,56]. Inactivated Japanese encephalitis and tick-borne encephalitis vaccines adjuvanted with alum induce strong early neutralizing antibody responses but show a gradual decline in antibody titers over time, necessitating periodic booster immunizations to maintain protective antibody levels [57].

Oil-in-water emulsions such as MF59 and AS03 act through distinct mechanisms. They stimulate local chemokine secretion (IL-6, and monocyte chemoattractant protein 1) and recruit antigen-presenting cells, leading to enhanced Tfh activation and affinity maturation beyond that achieved with alum [58,59,60]. In preclinical studies, these emulsions also promoted a more balanced Th1/Th2 profile and broader distribution of IgG subclasses, resulting in antibodies with improved functional quality [59,60,61]. Despite these advantages, emulsion adjuvants primarily increase the magnitude and functional breadth of early responses and still provide more limited support for prolonged germinal center reactions than replicating platforms do.

### 3.2. Novel Adjuvant Formulations Targeting B-Cell and T Helper Responses

Modern adjuvant discovery has focused on stimulating defined innate immune receptors that shape the magnitude and quality of B-cell selection within germinal centers. Toll-like receptor (TLR)-based adjuvants, such as CpG oligodeoxynucleotides (CPG ODN; TLR9) and monophosphoryl lipid A (TLR4), activate plasmacytoid dendritic cells and promote type I interferon and IL-12 production, favoring Th1 polarization and robust Tfh differentiation [62,63]. This cytokine milieu drives class switching toward IgG2a and IgG3 subclasses and enhances somatic hypermutation, resulting in higher affinity and improved Fc-mediated antibody function [62].

Saponin-based adjuvants, such as QS-21 and Matrix-M, act through inflammasome activation and antigen cross-presentation to expand Tfh and germinal center B-cell populations [64,65]. Evidence from preclinical studies in flaviviruses and other viral models suggests that these adjuvants can enhance antibody affinity and sustain higher titers over time, suggesting their utility in improving durability even when antigen doses are reduced. Emerging systems, such as cyclic dinucleotide STING agonists, activate follicular dendritic cells and promote extended antigen retention, which reinforces germinal center selection [66].

Lipid nanoparticle formulations used in mRNA vaccines provide both antigen delivery and intrinsic adjuvant activity through endosomal RNA sensing. This dual action leads to prolonged germinal center activity and efficient long-lived plasma cell development, as demonstrated in preclinical and early clinical studies of Zika and dengue mRNA vaccine candidates [67,68]. Collectively, these novel adjuvants demonstrate that targeted innate signaling can modulate antibody affinity, breadth, and persistence more effectively than conventional formulations.

### 3.3. Comparative Evaluation of Adjuvants Used in Flavivirus Vaccines

Head-to-head comparisons among flavivirus vaccine platforms illustrated clear differences in antibody durability and functional quality depending on adjuvant class. Alum-adjuvanted inactivated vaccines consistently produce high seroconversion rates but exhibit limited durability, with titers declining over several years [57,69]. In contrast, live attenuated vaccines, such as YF-17D or SA 14-14-2, generate long-lived immunity without external adjuvants because viral replication provides multifaceted innate stimulation that drives sustained Tfh activity and long-lived memory [43,70].

Preclinical data from flaviviruses and related viral vaccine models suggest that subunit- and DNA-based platforms may achieve improved antibody quality and durability when formulated with CpG, MPLA, or saponin adjuvants compared to alum alone [61,71]. Such formulations not only enhance germinal center longevity but also expand cross-neutralizing antibody repertoires that target conserved epitopes among flavivirus sero-complexes [72,73]. These effects are particularly relevant for dengue and Zika vaccines, where balancing the neutralization breadth and avoiding ADE remain key challenges.

A summary of these comparative outcomes is presented in Table 2, which outlines the major adjuvant classes, mechanisms of action, and their relative influence on antibody magnitude, affinity, and persistence in the host. Together, these results support the conclusion that rational adjuvant selection guided by systems immunology insights is central to designing next-generation flavivirus vaccines capable of eliciting high-quality, durable, and safe humoral immunity [29,74].

## 4. Effects of Antigen Design and Delivery Platforms

**Figure 1:**
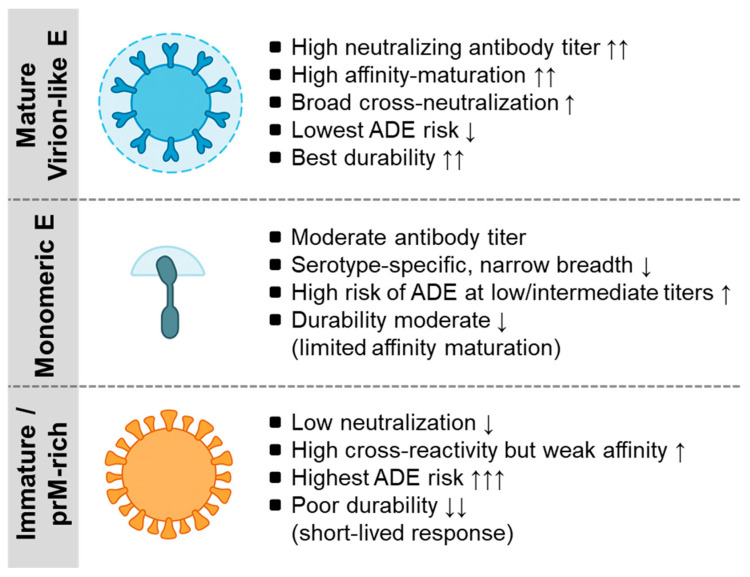
Antigen configurations of the flavivirus envelope and their immunological consequences. This schematic illustrates the three major forms of E protein antigen presentation and their associated humoral profiles. Mature virion-like E (e.g., YF-17D-like or SA14-14-2-like mature particles) expose organized quaternary epitopes that drive high neutralization potency, extensive affinity maturation, minimal ADE potential, and durable long-lived plasma cell responses. Monomeric E proteins (e.g., soluble recombinant E or EDIII subunits) have limited epitope organization and elicit moderate neutralization with narrower serotype-specific breadth and a higher ADE risk at low or intermediate antibody concentrations. Immature or prM-rich antigens (e.g., incompletely matured inactivated virions) display prM-shielded surfaces that generate weak neutralization, high cross-reactivity with low affinity, the highest ADE propensity, and short-lived antibody responses. Together, these three configurations illustrate how antigen conformation governs the quality, breadth, safety profile, and durability of antibodies across flavivirus vaccine platforms. For a detailed mechanistic discussion, see Section 4 and Section 5. ADE, antibody-dependent enhancement, EDE, envelope dimer epitope. The arrows will be defined as follows: ↑ increase, ↑↑ marked increase, ↑↑↑ strong increase, ↓ decrease, and ↓↓ marked decrease.

The structure and mode of flavivirus antigen delivery critically determine how the immune system perceives, processes, and retains antigenic information. Antigen conformation dictates epitope accessibility and the type of B-cell clones that dominate the response, whereas delivery platforms influence antigen persistence and the magnitude of germinal center activity. Therefore, preserving native quaternary structures and ensuring sustained antigen availability are central requirements for generating high-affinity and durable antibody responses (Figure 1). Modern vaccine designs incorporate mRNA, viral vectors, or nanoparticles to optimize these features and elicit persistent and broadly protective humoral immunity (Table 3). These platform-dependent differences reflect the first and fourth design principles, highlighting the importance of preserving quaternary E-dimer epitopes and optimizing antigen persistence to reinforce a durable and broadly neutralizing humoral immunity.

### 4.1. Structural Features of Flavivirus Envelope Proteins Influencing Antibody Quality

Flavivirus virions are composed of 180 copies of envelope glycoproteins arranged as antiparallel dimers on the viral surface [122]. This protein mediates receptor attachment and low pH-triggered membrane fusion, and its conformation governs the exposure of neutralizing epitopes. Antibodies that recognize quaternary epitopes spanning adjacent E protein dimers, such as EDE, demonstrate cross-neutralizing potential across dengue virus (DENV) serotypes and ZIKV [98,99].

In contrast, antibodies targeting the fusion loop or domain III often display strong serotype-specific neutralization but show limited breadth and carry increased ADE potential at sub-neutralizing concentrations [123,124]. These findings underscore the importance of preserving native quaternary E dimer organization to elicit antibodies with optimal quality and cross-protective potential. Structural vaccinology studies have shown that quaternary epitope exposure can be stabilized through engineered virus-like particles (VLPs) or pre-fusion-stabilized E proteins, which improve the neutralization breadth and reduce the targeting of non-protective regions [103,104]. Furthermore, the presence of prM protein in immature or partially mature particles can divert the immune response toward poorly neutralizing epitopes, emphasizing the need for antigen designs that minimize prM content and retain mature virion-like conformations [125,126].

### 4.2. Antigen Presentation and Delivery Mechanisms in Different Vaccine Platforms

Distinct vaccine platforms present flavivirus antigens in different structural and cellular contexts, influencing B-cell engagement and antibody persistence. Live-attenuated vaccines, such as YF-17D and SA 14-14-2 JEV, replicate transiently in vivo and provide sustained antigen exposure, a feature that supports prolonged germinal center activity and long-lived plasma cell formation [106,127]. Inactivated vaccines, which are safer for certain populations, present fixed antigens that may alter the E protein conformation and limit the induction of broadly neutralizing antibodies [128,129].

Recombinant subunit vaccines expressing soluble E or domain III proteins require potent adjuvants or multimeric presentation systems to elicit antibody responses owing to the limited capacity of monomeric antigens to crosslink B-cell receptors [130,131]. VLPs that mimic the native virion surface antibody repertoires are similar to those of natural infections and produce higher affinity responses than soluble proteins [132].

Nucleic acid-based platforms, such as mRNA vaccines, sustain antigen expression, promote prolonged germinal center activity, and promote efficient affinity maturation [96,133]. Similarly, viral vector vaccines using adenovirus or measles backbones express the E protein within host cells, supporting both humoral and T-cell immunity and providing balanced and durable protection [127]. Taken together, the balance between antigen stability, persistence, and structural fidelity determines whether the immune response favors high-avidity cross-neutralizing antibodies or short-lived strain-specific profiles.

### 4.3. Emerging Technologies for Improving Antigen Stability and Immune Persistence

Recent advances have aimed to enhance the stability and immunogenicity of flavivirus antigens through rational design and optimized delivery. Cryo-electron microscopy and computational modeling have identified key structural determinants of E protein flexibility, enabling targeted mutations that lock the protein in a pre-fusion conformation resistant to rearrangement [134,135,136]. This approach yielded modified E-dimer constructs that retained neutralizing epitopes, and the reduced exposure of cross-reactive regions exhibited limited neutralizing capacity.

Nanoparticle-based platforms further enhance antigen presentation by multimerizing E proteins or epitopes on scaffolds that replicate the virion geometry [137,138]. These structures efficiently engage B-cell receptors and promote strong Tfh responses, resulting in durable and high-avidity antibody production. Encapsulation of flavivirus antigens in lipid nanoparticles or biodegradable polymers can also improve thermostability and sustain antigen release, leading to extended germinal center activity over time [112,139].

Self-amplifying RNA- and replicon-based systems maintain antigen expression at lower doses and enhance both the magnitude and durability of antibody responses in dengue and Zika models [140,141]. Collectively, these emerging technologies support vaccine strategies that pair structurally stabilized antigens with delivery platforms capable of sustaining germinal center maturation to achieve broad, potent, and long-lasting humoral immunity across the flavivirus genus.

## 5. Cross-Reactivity and Immune Imprinting Among Flaviviruses

Cross-reactive antibody responses are hallmarks of flavivirus immunity, reflecting the high sequence and structural homology of the E protein across species [142]. These antibodies can provide cross-protection through recognition of conserved quaternary epitopes. Under sub-neutralizing conditions, they may also facilitate infection via Fcγ receptor-mediated uptake [143]. The balance between protection and enhancement depends on epitope specificity, the affinity maturation state, antibody concentration, and exposure history. These interactions underscore the fifth design principle, which incorporates baseline serostatus and immune imprinting patterns into the interpretation of vaccine-induced antibody breadth and safety.

### 5.1. Cross-Reactive Antibody Responses and Their Protective or Enhancing Roles

Flavivirus infections elicit a complex polyclonal antibody repertoire, some portion of which is cross-reactive with other species of the genus Flavivirus. Broadly neutralizing antibodies that target conserved quaternary epitopes, such as EDE, neutralize multiple DENV serotypes and exhibit partial cross-neutralizing activity against ZIKV [144,145]. These antibodies recognize conformational surfaces formed by adjacent E protein dimers, providing broad and robust Fc functionality [146,147].

In contrast, cross-reactive regions that exhibit limited neutralizing capacity, particularly fusion loop-directed or prM-associated epitopes, can promote the infection of Fcγ receptor-expressing cells, a process known as ADE [148,149]. Longitudinal studies in Nicaragua demonstrated a bell-shaped relationship between pre-existing antibody titers and severe dengue risk, with maximal enhancement occurring at intermediate titers, a pattern subsequently validated in multiple cohorts [24].

Dengue–Zika cross-reactivity is another aspect of this phenomenon. Antibodies elicited by prior dengue infection bind Zika E protein with high avidity but display variable neutralization potency and can enhance Zika infection of myeloid cells in vitro [148,150]. Nevertheless, high-affinity broadly neutralizing antibodies targeting conserved quaternary interfaces, including EDE or fusion loop-spanning regions, appear to be capable of conferring partial protection against these viruses.

### 5.2. Immune Imprinting and Its Impact on Vaccine Effectiveness

Immune imprinting, also referred to as the original antigenic sin, describes how the first flavivirus exposure biases subsequent antibody responses toward initially recognized epitopes [151]. This effect influences both infection outcomes and vaccine immunogenicity. In dengue vaccination, the history of primary infection determines the hierarchy of memory B-cell recall and the relative magnitude of type-specific versus cross-reactive antibodies. Clinical trials of the tetravalent chimeric YF dengue vaccine (CYD TDV, Dengvaxia) revealed that baseline seronegative recipients were at an elevated risk of severe disease following natural infection, whereas seropositive recipients demonstrated durable protective immunity [152].

Longitudinal sero-epidemiological studies during sequential epidemics in Central America and Southeast Asia have shown that Zika infection can modulate dengue outcomes, indicating imprinting across viral species [153]. This reciprocal relationship suggests that both natural and vaccine-induced priming can reprogram germinal center selection and shape antibody repertoire evolution during later exposures [154]. These findings the emphasize that pre-vaccination serostatus and local exposure patterns are key determinants of vaccine efficacy and safety.

### 5.3. Balancing Broad Protection and Safety in Vaccine-Induced Antibody Responses

Current vaccine strategies aim to favor epitopes associated with potent neutralization, while minimizing responses to regions prone to ADE. Structural vaccinology approaches that stabilize the E dimer conformation or mask the fusion loop help focus antibody responses on protective surfaces [155]. Parallel immunological studies have shown that adjuvants that promote Tfh cell activity and germinal center persistence, such as TLR- or saponin-based formulations, enhance affinity maturation and reduce the window of sub-neutralizing titers that permit ADE [156].

Incorporating baseline serological screening into dengue vaccination programs is essential to avoid enhancing the risk among seronegative individuals, and similar stratified approaches are being considered for future multivalent vaccines [157]. The next generation of flavivirus vaccines will likely integrate antigen design, adjuvant selection, and population serology to achieve broad protection with minimal risks. Among the various mitigation strategies discussed in this section, only baseline serostatus screening is currently implemented in real-world dengue vaccination programs, whereas antigen engineering, imprint-aware boosting schedules, and structure-guided epitope focusing remain investigational and context dependent. A summary of the representative cross-reactive and immune imprinting effects that influence flavivirus vaccine responses is presented in Table 4.

## 6. Strategies for Improving Antibody Maturation and Long-Term Maintenance

Achieving broad and durable antibody responses to flavivirus vaccines requires a coordinated strategy that optimizes the kinetics of B-cell activation, affinity maturation, and the maintenance of plasma cell and memory B-cell compartments. Recent human and animal studies have identified key determinants of antibody persistence, including booster timing, host factors such as age and immune history, and adjuvant or platform characteristics that sustain germinal center reactions [20,166,167,168]. Integrating these factors into vaccine design is essential to generate long-lasting immunity. The strategies detailed in this section expand on the third design principle, emphasizing approaches that sustain germinal center reactions and support long-lived plasma cell development to maintain durable antibody protection against pathogens.

### 6.1. Optimizing Booster Intervals and Heterologous Prime-Boost Regimens

The booster vaccination is a major driver of affinity, maturation, and protection. Inactivated JE vaccines show neutralizing antibody waning within 2–3 years, and periodic boosters restore protection by re-engaging memory B-cells and initiating new germinal center responses [107]. YF-17D demonstrates that a single dose can induce immunity that extends the presence of antigens associated with live attenuated replication [43,169].

Heterologous prime-boost regimes using distinct platforms can further enhance the antibody breadth and durability. Combining DNA or mRNA priming with live-attenuated or viral vector boosting increases germinal center output and promotes the selection of high-affinity B-cell clones [170,171]. In dengue and Zika models, sequential exposure to antigenically related but non-identical E proteins increased cross-neutralizing titers without broadening the enhancing antibody subsets, indicating that controlled heterologous priming can shape safe and effective immune imprinting [149,172]. These observations support the rational scheduling of booster intervals and cross-platform combinations to sustain antibody quality, while minimizing the risk of enhancement.

### 6.2. Host and Age-Related Factors Influencing Antibody Persistence

Host physiology and immune history strongly influence the humoral outcomes. Aging is associated with reduced germinal center size and diminished Tfh cell activity, leading to lower antibody affinity and shorter antibody half-life following vaccination [173,174]. Similar trends have been observed in infants, whose immature follicular responses and limited somatic hypermutation restrict antibody durability [175].

Pre-existing flaviviral immunity also affects viral persistence. Individuals with prior dengue exposure exhibit faster recall and higher avidity after JE or Zika vaccination than flavivirus-naïve subjects, which is consistent with antigenic imprinting that accelerates memory B-cell reactivation [162,176]. Nutritional and metabolic conditions further modulate antibody maintenance, as chronic inflammation and altered cytokine balance can reduce the availability of survival niches for long-lived plasma cells in the bone marrow [177]. Therefore, tailoring vaccine formulations and booster schedules to specific age groups and baseline serostatuses is essential to ensure durable population-level protection.

### 6.3. Approaches to Sustain Germinal Center Activity and Memory B-Cell Formation

Sustained germinal center activity supports the continuous selection of high-affinity clones and development of long-lived plasma cells. Vaccine formulations that prolong antigen availability or activate innate pathways that support Tfh cell functions can enhance this process. Adjuvants, such as CpG ODN and MPLA, induce IL-21 and interferon gamma (IFN-γ) production, maintain follicular dendritic cell networks, and support persistent germinal centers [178,179]. Nanoparticle and mRNA platforms extend antigen expression, resulting in durable plasma cell output and strong memory B-cell recall [180,181].

In murine flavivirus models, follicular dendritic cell activation and BAFF/APRIL signaling are critical for the survival of long-lived plasma cells, and approaches that preserve these cytokines enhance antibody half-life [182,183]. Experimental strategies, including cyclic dinucleotide STING agonists or saponin-based adjuvants, sustain germinal center activity for extended periods, yielding antibodies with greater affinity and improved Fc functional profiles [184]. Together, these findings indicate that combining platforms that provide prolonged antigen presence with adjuvants that maintain Tfh cell support and stromal signaling is central to sustaining long-term humoral immunity during flavivirus vaccination.

## 7. Future Perspectives and Vaccine Design Considerations

Recent advances in immunology, adjuvant chemistry, and structural vaccinology have transformed flavivirus vaccine development into a data-driven, precision-focused field. The next generation of vaccines aims to combine rational antigen design with optimized adjuvants and delivery systems to elicit antibodies with high affinity, functional diversity, and long-term persistence [129,185,186,187]. Insights gained from the dengue, JE, YF, and Zika vaccines provide a foundation for future strategies to achieve both breadth and safety in diverse populations. These developments collectively highlight the importance of integrating antigen stability, innate activation, and population-specific considerations into future vaccine designs. These forward-looking strategies integrate multiple design principles introduced in the Introduction, particularly those concerning epitope-focused antigen engineering, optimized antigen persistence, and incorporation of serostatus-driven immune imprinting.

### 7.1. Integration of Adjuvant and Antigen Design for Improved Humoral Immunity

One promising direction in flavivirus vaccinology is the coordinated optimization of the antigen structure and innate immune stimulation. Modern adjuvants not only boost antibody magnitude but also modulate germinal center dynamics and B-cell selection, shaping the qualitative features of humoral immunity [53,188].

Structure-guided antigen engineering has identified conserved quaternary epitopes on the envelope dimer interface that can be stabilized by targeted mutations or scaffold presentation to maintain neutralization-sensitive conformations [98,189].

Combining stabilized immunogens with potent adjuvants, such as saponins or TLR-based formulations, can prolong germinal center activity and favor the development of high-affinity cross-neutralizing antibodies with reduced likelihood of enhancement [164,190]. These integrated approaches support the coordinated activation of B-cells, Tfh cells, and long-lived plasma cell formation, thereby improving the durability and safety of the humoral responses.

### 7.2. Lessons from Flavivirus Vaccine Platforms for Universal Vaccine Development

Decades of experience with live attenuated, inactivated, and recombinant flavivirus vaccines have generated insights that have extended beyond a single viral genus. The durable immunity elicited by YF-17D indicates that sustained antigen expression combined with robust innate activation can provide near-lifelong protection [43,70]. In contrast, the variable outcomes observed with the tetravalent dengue vaccine (CYD-TDV) emphasize the complexity of immune imprinting and the importance of assessing baseline serostatus when evaluating vaccine performance [172,191].

These lessons inform the design principles of next-generation platforms that employ self-amplifying RNA, viral vectors, or nanoparticle-based delivery to achieve controlled antigen persistence and balanced innate and adaptive activation [141]. Cross-platform and heterologous prime-boost regimens can be tailored for distinct epidemiological settings, ensuring that responses remain durable and broad across flavivirus species [154]. The integration of these approaches provides a foundation for developing pan-flavivirus vaccines and may inform universal vaccine approaches for related viral families, including alphaviruses and bunyaviruses [192].

### 7.3. Outlook for Next-Generation Flavivirus Vaccines

Future vaccine development will increasingly rely on predictive analytics and high-dimensional immune profiling to define correlates of durable protection. Systems vaccinology approaches integrating transcriptomics, B-cell receptor repertoire sequencing, and machine learning can identify molecular signatures associated with long-lived plasma cell formation and high-quality antibody responses [193,194]. These insights will enable the rational selection of adjuvants and structural immunogens that maximize protective breadth while maintaining safety across diverse host immune backgrounds.

Population-tailored vaccination strategies have become increasingly important. Adjusting booster intervals, selecting appropriate adjuvants, and adapting platforms according to age, sex, and baseline serostatus can enhance both the durability and equity of vaccine impact [127].

Advances in artificial intelligence for epitope prediction and computational immunogen design have accelerated the development of optimized flavivirus antigens capable of eliciting broad neutralization with minimal risk of ADE [195]. Collectively, these developments illustrate the transition from empirical vaccine discovery to precision vaccinology guided by structure, systems biology, and population-level data. A concise summary of emerging strategies and anticipated directions for flavivirus vaccine development is presented in Table 5, which synthesizes how these approaches align with the design principles introduced at the end of the Introduction.

**Table 5:** Future strategies for improving flavivirus vaccine-induced antibody response.

Strategic Area	Emerging Concept or Approach	Expected Immunologic Impact	Development Stage or Outlook	Refs.
Structure-guided epitope optimization	Stabilization of E-dimer or mosaic epitopes that preserve cross-neutralizing structures	Focuses antibody response on protective epitopes and lower enhancement risk	Preclinical studies and Phase 1 early clinical testing	[98,158,196]
Adjuvant and antigen co-engineering	Incorporation of PRR agonists or saponin-based adjuvants into mRNA or nanoparticle platforms	Sustains germinal center activity and enhances affinity maturation	Advanced preclinical studies and Phase 1–2 clinical evaluation	[164,190,197]
Self-amplifying RNA and replicon platforms	Extended antigen expression with low-dose formulations	Increases magnitude and duration of antibody response	Phase 1 clinical trials	[198,199,200]
Pan-flavivirus vaccine design using conserved scaffolds	Use of shared E dimer and fusion loop epitope scaffolds to support broad cross protection	Enables cross serotype and cross species immunity across dengue, Zika, and JEV	Computational design with mechanistic inference	[26,101,158]
Systems vaccinology and immune profiling	Integration of omics signatures and antibody repertoire sequencing to define correlates of protection	Predicts antibody quality and durability for vaccine optimization	Research-stage integration with exploratory clinical programs	[201,202,203]
Population-tailored vaccination strategies	Adjustment of prime boost intervals and formulations based on serostatus and age	Maximizes protective efficacy while reducing enhancement risk	Program-level evaluation in endemic populations (observational evidence)	[152,157,204]
Combination vector or heterologous prime-boost approaches	Sequential mRNA, viral vector, or subunit vaccination	Promotes balanced B and T cell immunity with improved durability	Preclinical and translational-stage development	[205,206,207]
Artificial intelligence assisted immunogen design	Machine learning based prediction of stable neutralizing epitopes and escape variant targets	Accelerates design of rationally optimized immunogens	Computational modeling with mechanistic inference	[208,209,210]

The evidence tiers in this table are standardized using preclinical murine or NHP studies, phase 1–2 early clinical trials, phase 3 clinical data, and computational or mechanistic inference, consistent with the terminology used throughout the manuscript. PRR, pattern recognition receptor.

## 8. Conclusions

Flavivirus vaccine development has transitioned from empirical formulations to a mechanistic understanding of how antigen structure, innate immune activation, and host history shape the quality and durability of antibody responses. Decades of work with live attenuated and inactivated vaccines have demonstrated that long-term sero-protection is achievable when antigen integrity and immune stimulation are optimally balanced. The YF-17D and JE SA 14-14-2 vaccines remain key examples of how replication-driven innate signaling and strong germinal center induction generate durable, high-avidity antibodies [70,211]. In contrast, dengue vaccination highlights the dual behavior of cross-reactive antibodies, which may mediate either broad protection or antibody-dependent enhancement depending on antibody concentration, epitope specificity, and affinity maturation state [212,213].

Modern immunology and structural biology have expanded the correlates of protection beyond neutralizing titers. Antibody affinity, subclass distribution, and Fc effector functions are now recognized as critical determinants of effective immunity. Broadly neutralizing antibodies that target conserved quaternary epitopes on the envelope dimer provide structural templates for rational immunogen design [214]. Antigen engineering approaches that stabilize the pre-fusion E conformation or display key epitopes on virus-like or nanoparticle scaffolds can directly respond to protective surfaces while limiting the exposure to enhancing epitopes. Parallel progress in adjuvant research has demonstrated that engaging pattern-recognition receptor pathways, including TLR and STING signaling, can sustain germinal center activity and promote long-lived plasma cell formation, supporting improved antibody affinity and persistence [215].

However, despite these advances, several important challenges remain. Immune imprinting, baseline serostatus, and variation in vector exposure continue to influence vaccine effectiveness, particularly in dengue- and ZIKV-endemic regions. Validated correlates of durable protection and long-term field data are needed to interpret laboratory-defined immune signatures and inform evidence-based vaccination schedules. Furthermore, thermostable, low-cost, and easily deployable vaccine formulations are essential for ensuring equitable access in regions where flavivirus transmission remains a major public health concern. Addressing these priorities will require a closer integration of immunology, epidemiology, and implementation sciences.

Future vaccine development is expected to increasingly rely on computational modeling, systems vaccinology, and artificial intelligence to predict immunogenicity and guide structural antigen optimization [216]. Combining structure-guided epitope mapping with coordinated adjuvants and platform engineering offers a path toward precision vaccines that can achieve high-quality, long-lasting, and cross-protective antibody responses. Population-tailored strategies that consider age, immune background, and prior flavivirus exposure may further enhance its safety and effectiveness.

Collectively, these developments indicate a shift toward precision vaccinology, which integrates molecular, computational, and population-level insights to achieve broad, durable, and safe humoral immunity. Next-generation flavivirus vaccines are expected to provide sustained protection against dengue, Zika, JE, and other emerging viruses, and contribute to the broader goal of designing universal vaccines for complex RNA viruses. Ensuring global accessibility and equitable deployment ultimately define the success of scientific advances.

## Figures and Tables

**Table 2:** Comparative effects of classical and novel adjuvants on antibody responses to flavivirus vaccines.

Adjuvant Class	Mechanism Target	Immune Profile	Effects on Antibody Quality	Durability Titer	Representative Flavivirus Context	Refs.
Alum	NLRP3 inflammasome activation; antigen depot	Predominantly Th2; limited Tfh	Increases antibody magnitude but limited affinity maturation	wanes over 2–3 years	Inactivated JE, TBEV, WNV vaccines	[75,76,77,78,79]
MF59 (oil-in-water emulsion)	Enhanced APC recruitment and cytokine induction	Balanced Th1/Th2; improved Tfh	Enhances avidity and broad subclass distribution	up to 5 years in available studies	WNV and JE experimental vaccines	[61,80,81]
AS03	Innate sensor activation, IL-6 and cytokine induction	Balanced Th1/th2 with robust Tfh	Improves avidity and functional diversity	≥3 years in clinical follow up	DENV and ZIKV subunit studies	[82,83]
CpG ODN (TLR9 agonist)	TLR9 activation of plasmacytoid DCs, IL-12 and IFN induction	Strong Th1 and Tfh	Enhances class switching & affinity maturation	anticipated from platform mechanism	JEV and WNV DNA vaccine studies	[84,85,86,87]
MPLA (TLR4 agonist)	NF-κB and TRIF signaling	Th1-skewed, synergizes with alum	High-avidity antibodies; enhanced Fc-mediated functions	wanes over 2–3 years	DENV and YF envelope antigen studies	[87,88,89]
QS-21 (saponin)	ISCOM formation; antigen cross-presentation	Balanced Th1/Th2; expanded Tfh	High-affinity, polyfunctional antibodies	Sustained long-term titers (≥3 years)	DENV and Matrix-M flavivirus platforms	[89,90,91]
STING agonists	Cytosolic DNA sensing; STING–IRF3 pathway	Strong Th1/Tfh; DC activation	Enhances affinity maturation & cross-neutralization	anticipated from platform mechanism	DENV and ZIKV preclinical vaccines	[92,93,94]
Lipid nanoparticle (LNP)	RNA sensing (TLR7/8); antigen expression	Th1-skewed; strong Tfh and GC	High-avidity antibodies; balanced subclass	anticipated from platform mechanism	mRNA ZIKV vaccines	[30,52,95,96]
Live-attenuated platform (self-adjuvanted)	Multifaceted innate sensing (RIG-I, TLR3, TLR7)	Balanced Th1/Tfh; strong GC	High-avidity, polyclonal antibodies	>20 years in clinical follow up	YF-17D, JE SA 14-14-2	[43,97]

Durability descriptors follow the standardized terminology used throughout the manuscript. Evidence tiers are harmonized using the categories Phase 3 clinical data, preclinical murine or NHP data, observational serology, and mechanistic inference, consistent with the terminology used throughout the manuscript. Th, helper T cell; Tfh, follicular helper T cell; GC, germinal center; DC, dendritic cell; LNP, lipid nanoparticle; ISCOM, immune stimulating complex; NF-κB, nuclear factor-kappa B.

**Table 3:** Antigen and delivery platform strategies enhance antibody durability.

Platform or Antigen Type	Structural or Mechanistic Features	Effect on Antibody Quality (Affinity/Breadth)	Durability Tier	Representative Flavivirus Context	Refs.
Native virion-like E (quaternary epitope preserving)	E dimers maintain authentic virion-like quaternary organization	Induces high-affinity, cross-neutralizing antibodies recognizing EDE and domain III	>20 years in clinical follow up	Dengue and Zika native E dimer constructs	[98,99,100]
Prefusion-stabilized E protein mutants	Engineered mutations lock E in prefusion conformation	Focuses B-cell responses on neutralizing epitopes; reduces non-neutralizing reactivity	≥4 years in clinical trials so far	Stabilized DENV and JEV E proteins	[98,101,102]
VLPs	Multivalent epitope displays mimicking mature virion surface	Enhances BCR cross-linking and Tfh activation; increases antibody avidity	anticipated from platform mechanism	Zika and DENV VLP vaccine candidates	[103,104]
Live-attenuated platform	Limited replication provides prolonged antigen exposure & innate activation	Generates high-avidity polyclonal antibodies with road diversity	>20 years in clinical follow up	YF-17D, SA14-14-2 JE	[105,106]
Inactivated whole-virus vaccines	Chemical inactivation may distort E conformation	Produces serotype-specific antibodies with limited breadth	wanes over 2–3 years	IXIARO (JE), TBEV vaccines	[13,107,108]
Recombinant subunit (E monomer or domain III)	Soluble antigens present only monomeric epitopes	Elicits moderate titers; limited affinity maturation unless adjuvanted	anticipated from platform mechanism	Dengue E and domain III vaccines	[109,110,111]
mRNA platform	Sustained intracellular antigen expression in LNPs; innate RNA sensing	Drives robust GC reactions and high-avidity antibody production	≥4 years in clinical trials so far	mRNA Zika and dengue candidates	[52,112,113]
Viral vector platform (adenovirus, measles, VSV)	Intracellular expression of E proteins mimicking infection	Balanced humoral and T-cell responses; strong neutralization	≥4 years in clinical trials so far	Adenoviral DENV and measles-ZIKV constructs	[114,115,116]
saRNA/replicon systems	RNA replication prolongs antigen expression at low doses	Enhances GC responses and cross-serotype breadth	anticipated from platform mechanism	saRNA dengue and Zika vaccine candidates	[117,118,119]
Nanoparticle scaffolds or polymer-encapsulated antigens	Multimerized epitope display; sustained release	Enhances BCR cross-linking and Tfh responses	anticipated from platform mechanism	Flavivirus VLP or polymeric constructs	[120,121]

Durability descriptors follow the standardized terminology used throughout the manuscript. Evidence tiers are harmonized as Phase 3 clinical data, preclinical murine or NHP data, observational serology, and mechanistic inference, consistent with the terminology used throughout the manuscript. EDE, envelope dimer epitope; GC, germinal center; Tfh, follicular helper T cell; BCR, B cell receptor; LNP, lipid nanoparticle; saRNA, self-amplifying RNA.

**Table 4:** Cross-reactivity and immune imprinting effects of the flavivirus vaccination.

Phenomenon	Prior Exposure or Baseline State	Dominant Antibody Features	Observed Outcome	Vaccine or Epidemiologic Context	Refs.
Cross neutralization through quaternary epitope recognition	Prior dengue infection or vaccination with antigens that preserve E dimer geometry	Broadly neutralizing antibodies targeting the envelope dimer epitope with high avidity	Protection across dengue serotypes and partial Zika cross protection	Human monoclonal antibody and structural studies (mechanistic inference)	[26,27,158]
Enhancement at intermediate antibody titers	Previous dengue infection with waning or incomplete neutralizing titers	Cross reactive antibodies with strong Fc gamma receptor binding but low neutralization potency	Increased risk of severe dengue during reinfection	Prospective Nicaraguan pediatric cohort (observational serology)	[23,159]
Dengue to Zika cross reactivity	Prior dengue exposure before Zika introduction	Antibodies recognizing Zika envelope epitopes with variable neutralization	In vitro enhancement and partial protection in vivo	Human sera from 2016 Zika epidemic (observational serology)	[148,158]
Immune imprinting shaping vaccine outcomes	Seronegative or monotypic dengue infection at baseline	Recall of original epitope hierarchy with limited breadth after vaccination	Reduced efficacy and higher hospitalization risk in seronegative vaccinees	CYD-TDV Phase 3 trials (Phase 3 clinical data)	[152,160,161]
Post Zika increase in dengue severity	Zika infection followed by dengue exposure	Cross reactive antibodies with altered Fc function	Higher incidence of severe dengue following Zika epidemics	Population-level analyses in Central America (observational epidemiologic evidence)	[153,162]
Mitigation through antigen focus and enhanced maturation	Use of structurally stabilized E antigens and potent adjuvants	High avidity neutralizing antibodies with reduced non neutralizing responses	Broader and safer protection in preclinical and early clinical studies	Dengue and Zika subunit or mRNA vaccine development (preclinical and early phase clinical data)	[98,163,164,165]

The evidence tiers in this table follow the standardized terminology used throughout the manuscript, including Phase 3 clinical data, preclinical murine or NHP studies, observational serology, and mechanistic inference, consistent with the terminology used throughout the manuscript. Fc, fragment crystallizable region of the antibody.
